# Knowledge graph-based intelligent data management and information innovation service model for university library systems

**DOI:** 10.1371/journal.pone.0341307

**Published:** 2026-01-16

**Authors:** Fanru Liu

**Affiliations:** Dalian University of Technology Library, Dalian University of Technology, Dalian, Liaoning, China; Centro de Investigacion en Ciencias de Informacion Geoespacial AC (Research Center on Geospatial Information Sciences), MEXICO

## Abstract

With the rapid development of digitalization, university libraries find themselves under great pressure in adapting to data management, whereas traditional management has limitations in meeting personalized needs for information. This study constructs a knowledge graph-based intelligent data management and information innovation service model for university library systems, which adopts a hierarchical design philosophy encompassing five core layers: data source layer, data processing layer, knowledge construction layer, service application layer, and user interaction layer. By integrating multi-source heterogeneous data resources and establishing a unified knowledge representation framework, the model facilitates semantic organization as well as automatic management of library information. The model employs dynamic fusion methods combining large language models and graph embedding to address heterogeneous data integration challenges, while leveraging knowledge graph semantic association capabilities to provide precise personalized information recommendation services. A systematic evaluation conducted for a period of six months shows that score for an user experience is 4.40, pointing to an improvement of 45.2% from 3.03, with accuracy in search results increasing by 41.1%, as well as enhancement of service quality and learning effectiveness by 32.5% and 41.7% respectively, and all 16 technical indexes having met and exceeded set standards. This study proposes a realistic solution to help university libraries deal with challenges brought by big data, which also facilitates intelligent service transformation for university libraries.

## 1. Introduction

With the profound advancement of the digital era, university libraries are facing unprecedented challenges to address different needs for information from library users. Academic libraries currently face pervasive issues in research data management practices, including difficulties in data integration and low service efficiency, as traditional management paradigms can no longer accommodate users’ increasingly sophisticated personalized information requirements [1]. Content analysis of academic library services shows that although most academic libraries have increased their electronic collections, there are still gaps in offering intelligent, user-centered retrieval and recommendation tools, which are capable of satisfying information needs in an optimized manner [2]. Particularly when processing large-scale digital datasets, university libraries encounter multiple challenges including technological barriers, insufficient personnel competencies, and the absence of effective data governance frameworks [3]. These issues not only compromise library service quality but also constrain their pivotal role in supporting academic research and educational activities.

Knowledge graphs, as an emerging technology for knowledge representation and organization, offer innovative approaches to addressing library data management challenges. Systematic research on knowledge graph construction and application in the educational domain demonstrates that knowledge graphs can effectively integrate multi-source heterogeneous data, achieve structured knowledge representation and intelligent reasoning, thereby significantly enhancing educational resource utilization efficiency [4]. The successful application of large language model-based knowledge graph construction methods in oral history archival resource processing further validates the great potential of knowledge graph technology in the field of bibliographic information organization [5]. Educational knowledge graph construction not only improves personalized learning experiences but also provides robust technical support for curriculum design, concept mapping, and educational content recommendation systems.

The dissemination of scholarly publications, as well as digital resources, has catalyzed opportunities as well as challenges for libraries in meeting diverse users information needs more effectively. Currently, university library users vary from undergraduates in search of basic knowledge to faculty members engaged in highly specialized research, characterized by different usage patterns of information as well as different standards of service demand [6]. Examining digital library development from a user service perspective reveals that traditional service models exhibit evident limitations when addressing information retrieval, knowledge discovery, and personalized recommendation needs in big data environments [7]. Practical case studies indicate that constructing effective library research data management service systems requires comprehensive consideration of multiple dimensions including user characteristics, service infrastructure, and collaborative mechanisms [8]. The implementation experience of Peking University Library in open science data management demonstrates that establishing systematic data management frameworks and service processes can effectively promote open science practices and data sharing [9]. For libraries, the challenge is not only how many resources they have at hand, but also understanding, anticipating, and responding to individuals’ needs.

This study is dedicated to constructing a knowledge graph-based intelligent data management and information innovation service model for university library systems. An information innovation service model refers to a service paradigm that aims to convert conventional information delivery into knowledge-based intelligent services by focusing more on proactive discovery, as opposed to passive discovery by using advanced technologies. Through integrating multi-source heterogeneous data resources and establishing a unified knowledge representation framework, this model achieves semantic organization and intelligent management of library information resources. Based on knowledge graph reasoning capabilities and path analysis methods, the model can provide users with precise personalized information recommendation services, significantly enhancing information retrieval efficiency and user satisfaction. Simultaneously, by introducing intelligent service strategies and innovative application scenarios, this model provides theoretical guidance and technical pathways for university library digital transformation and service model innovation. The value of this study is to offer possible solutions for university libraries to provide personalized, intelligent information services to better adapt to the needs of current university users, as well as provide valuable references for related theoretical studies.

## 2. Literature review

This study undertook a systematic literature search in Web of Science, Scopus, IEEE Xplore, SpringerLink, ScienceDirect, and Google Scholar, using English keywords such as “knowledge graph + information management,” “knowledge graph + digital library,” “layered architecture + knowledge graph,” “data fusion + heterogeneous data,” and “multi-layer knowledge graph,” as well as Chinese equivalents. The initial search created approximately 150 publications, which were reduced to 47 relevant publications after screening.

### 2.1 Research progress on intelligent information service systems in libraries

Compared to earlier developmental phases, the application of open-source software in library systems has gradually increased, while AI-driven chatbots and text generation services have exerted profound impacts on educational institutions and libraries [10,11]. The application of virtual reality and eye-tracking technologies in intelligent library information service systems provides new possibilities for enhancing user experience, thereby achieving more precise information recommendations and service optimization [12]. The construction of library intelligent service systems from an artificial intelligence perspective emphasizes the deep integration of technology and educational services, transforming traditional library service models and user interaction modalities through intelligent approaches [13]. Integrated library systems, as the core technological infrastructure of modern libraries, have evolved from traditional manual operations to highly automated enterprise resource planning systems, establishing a crucial foundation for constructing knowledge graph-based intelligent service systems.

### 2.2 Utilization of knowledge graphs in information management

Knowledge graphs, as a structured knowledge representation methodology, demonstrate tremendous application potential and developmental prospects in the information management domain. The successful application of large language model-enabled knowledge graph construction technologies in the framework materials field illustrates that through analyzing over 100,000 academic articles, it is possible to construct large-scale knowledge graphs containing 2.53 million nodes and 4.01 million relationships, effectively supporting the development of data retrieval, mining, and intelligent question-answering systems [14]. The application scenarios of knowledge graphs are continuously expanding, encompassing multiple domains including healthcare, financial services, and retail commerce, with particularly prominent performance in search engine optimization, recommender system construction, and natural language processing [15]. Recent developments in large language model-based knowledge graph construction tools demonstrate that by introducing new functionalities such as community summarization, parallel retrievers, and extended model support, it is possible to significantly enhance the efficiency and quality of knowledge graph construction from textual sources. Research on knowledge graph applications in entity retrieval indicates that through integrating multi-relational heterogeneous graph structures, information retrieval accuracy and relevance can be effectively improved [16]. These research achievements provide essential theoretical foundations and technical references for the application of knowledge graphs in library information management.

Typically, knowledge graphs utilize a layer-based, modular structure which is able to support data acquisition, graph construction, mining, processing, as well as providing services, using well-defined layers for data storage, processing, as well as provision of services [17]. Layering is originated from traditional software engineering; here, software is divided into hierarchical system elements which possess well-defined responsibilities [18]. In the case of knowledge graph-based recommender systems, this layering makes complex knowledge reasoning as well as provision of services into independent modules, thus increasing flexibility and scalability [19]. This approach has also proven to be successful in practical applications, for example, in digital libraries, as it allows for integration of multiple heterogeneous data sources, implementing hybrid recommendation approaches [20]. From synthesizing previous work, most of the knowledge graph solutions follow a four-layer modular structure consisting of a data layer, data processing layer, knowledge graph layer, and service layer, which together constitute a complete recommendation system. Given that many applications also involve data retrieval from domain-specific databases, this study further refines this four-layer framework by distinguishing the service layer into an intelligent service layer and an application layer, thus making possible data querying and interfacing for more comprehensive recommendation functions.

### 2.3 Digital transformation and innovative service paradigms in academic libraries

Current academic library trend analyses and artificial intelligence library service innovation conceptual frameworks indicate that libraries are rapidly expanding digital collections, emphasizing user welfare and AI technology applications, with focus on achieving service innovation and value creation through technological advancement [21,22]. Digital transformation readiness research and multidisciplinary perspective analyses reveal that academic preparedness for digital transformation is closely correlated with information literacy outcomes, with digital transformation emerging as a significant cross-disciplinary concern [23]. Literature reviews on the current state of digital transformation research and research data management challenges in citizen science projects indicate that scientific output related to digital transformation exhibits exponential growth, while libraries play crucial roles in supporting cross-disciplinary collaboration and data management [24].

Digital transformation in academic libraries is an imperative for addressing challenges in this current age of digitization. There is evidence to suggest that systematic technological training is an effective means of bringing about this transformation, although libraries are faced with challenges of constraints in financial, technological, as well as personnel skill development processes [25,26]. The fundamental elements of prevailing transformation models include digital culture, competent library professionals, resources for providing services, as well as technologies, in which digital reading is an essential pathway that impacts library services positively in relation to user’s satisfaction [27]. Libraries are rapidly expanding their digital collections and prioritizing the application of artificial intelligence alongside user well-being, leveraging technological advances to achieve service innovation and value creation [21,22]. Readiness for digital transformation is inextricably connected with information literacy outcomes and has become an important focal point across multiple disciplines [23]. Scientific output in this field has grown exponentially, and libraries are essential components in facilitating inter-disciplinary interactions for data research management [24].

In data fusion as well as the use of library knowledge graphs, data integration is critical to enhancing recommendations in a multifaceted manner. A hybrid recommendation model that fused heterogeneous data from social relationships, ratings, and review information in a comprehensive manner at the data source level, as opposed to combining results, was also proposed by Ji and other colleagues [28]. Heterogeneous information networks offer a comprehensive framework to incorporate multiple types of auxiliary information in an organic manner, which greatly benefits most current recommendation algorithms in terms of performance as well as interpretability [29]. For library applications, intelligent recommendation models combining fuzzy logic and deep learning algorithms are capable of dealing with uncertainty and vagueness in users’ preferences, improving prediction accuracy by up to14.6% when recommending personalized results through using collaborative filtering techniques [30]. The probabilistic keyword models, which integrated circulation data with keyword-attribute data, overcame data sparsity issues effectively [31]. Furthermore, optimized Hidden Markov Models, together with weight fuzzy rankings based upon temporal weightages and similarity in relation to the department, facilitated accurate subject classification suggestions [32]. Collectively, all of these methods are pushing library recommendation systems from matching paradigms to knowledge-based, semantically informed deep integration approaches, but most of existing work is still contained in offline dataset validations, without much practical application in libraries.

Based upon the literature reviewed above, this study proposes two fundamental research questions:

Research Question 1: How can an effective knowledge graph-based intelligent data management model for university libraries be constructed?
Research Question 2: What are the effects of knowledge graph-based intelligent data management models on enhancing university library information service quality and user experience?

## 3. Description of the knowledge-graph-based data management and service model

### 3.1 Architecture design for knowledge graph-based intelligent data management

**Fig 1:**
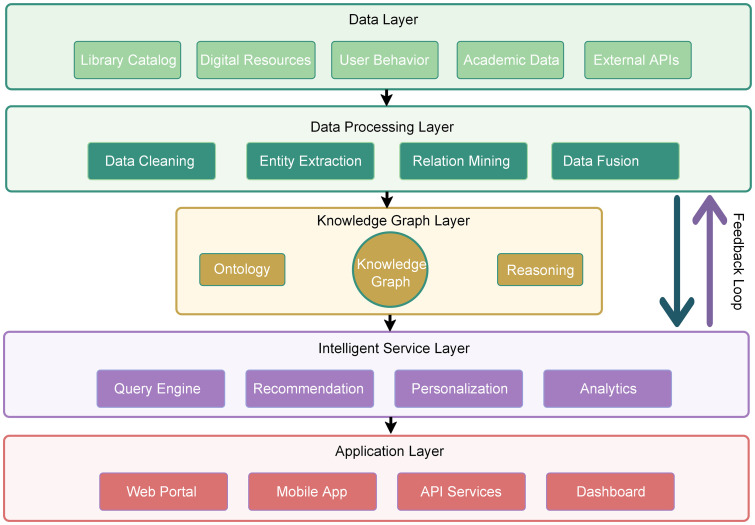
Five-layer architecture diagram for knowledge graph-based library intelligent data management. The architectural structure consists of five layers (color-coded): Data Layer (light green) for integration of multiple data sources, Data Processing Layer (dark green) for ETL processes, Knowledge Graph Layer (gold) for ontology-based knowledge development; Intelligent Service Layer (purple) for AI-based services, Application Layer (coral) for user interfaces. Black arrows denote data flow; purple demonstrates feedback for improvement.

Based on the practical requirements for intelligent services in university libraries, this study proposes a knowledge graph-based intelligent data management architecture. This architecture combines the comprehensive advantages of big data fusion technologies with knowledge graphs [33], adopting a hierarchical design philosophy that encompasses five core layers from bottom to top: data source layer, data processing layer, knowledge construction layer, service application layer, and user interaction layer, as illustrated in Fig 1. The data source layer is responsible for integrating bibliographic data, user behavior data, digital resource metadata from within the library, as well as external academic database information, establishing unified access for multi-source heterogeneous data. The data processing layer transforms raw data into structured knowledge representation forms through technologies including data cleansing, format conversion, entity recognition, and relationship extraction. The knowledge construction layer, based on ontology modeling and semantic reasoning technologies, employs knowledge graph-supported information fusion methods [34] to construct multi-dimensional knowledge graph networks encompassing books, authors, disciplines, user interests, and other dimensions. The service application layer, supported by knowledge graphs, implements innovative service functionalities including intelligent retrieval, personalized recommendation, and academic association discovery. The user interaction layer provides diversified service interfaces, supporting multiple access modalities including web-based, mobile, and API interfaces.

Systematic data flow between layers is achieved through standardized data interfaces and processing mechanisms, with feedback optimization loops established for continuous system performance improvement. Data flow between layers is implemented by using the W3C Linked Data stack, consisting of RDF 1.1 as an abstract data model expressed through using JSON-LD 1.1, OWL 2 for domain modeling, SKOS for domain vocabularies, SPARQL 1.1 for query, and SHACL for constraints, whereas interrepository interoperability is facilitated by OAI-PMH for harvesting, as well as by SRU/CQL for standards-based search [35]. The entire architecture adheres to current trends in data management and architectural development [36], leveraging the semantic association characteristics of knowledge graphs to break through traditional library information silos, achieving intelligent transformation from data management to knowledge services.

The library knowledge graph is formally defined as G = (E, R, T), where E = {e₁, e₂,..., eₙ} represents the entity set containing multi-type entities including books, authors, users, and disciplines; R = {r₁, r₂,..., rₘ} represents the relation set encompassing semantic associations such as borrowing, citation, and disciplinary affiliation; and T = {(eᵢ, rⱼ, eₖ)} represents the knowledge triple (see S1 File) set constituting the fundamental structural units of the graph. Through entity embedding function φ: E → ℝᵈ and relation embedding function ψ: R → ℝᵈ, the system maps discrete symbolic spaces to continuous vector spaces, where d denotes the embedding dimensionality. The data source layer is responsible for integrating internal library bibliographic data, user behavior data, digital resource metadata, and external academic database information, establishing unified access for multi-source heterogeneous data. The data processing layer employs technologies including data cleansing, format conversion, entity recognition, and relationship extraction to transform raw data into structured knowledge representations. The knowledge construction layer, based on ontology modeling and semantic reasoning technologies, constructs knowledge graph networks encompassing multi-dimensional semantic associations.

### 3.2 Multi-source heterogeneous data fusion and knowledge extraction methods for libraries

Multi-source heterogeneous data fusion and knowledge extraction constitute critical technological components in constructing intelligent management architectures. Addressing the complex scenario where multiple data formats coexist in library environments, including MARC records, digital resource metadata, and user behavior logs, this study employs a dynamic fusion method based on large language models and graph embedding [37] to achieve effective integration of heterogeneous data sources. This method constructs a unified data schema mapping mechanism that standardizes metadata of different formats into RDF triple representations T = {(eᵢ, rⱼ, eₖ)}, where eᵢ and eₖ represent source and target entities respectively, and rⱼ represents the semantic relationship between them, thereby resolving data silo problems in traditional library systems.

During the knowledge extraction process, the system employs intelligent fusion technologies [38], combining library domain ontologies and semantic rules to automatically identify key information elements such as bibliographic entities, author relationships, and subject classifications through the heterogeneous data fusion function F(D₁, D₂,..., Dₙ) = Σᵢ = ₁ⁿ αᵢDᵢ, where αᵢ represents the weight coefficient for each data source and satisfies Σαᵢ = 1. The system proposes a confidence evaluation mechanism Conf(t) = w₁ × Sim(t, KB) + w₂ × Freq(t) + w₃ × Source(t), where Sim(t, KB) is the semantic similarity value between triple t and an available knowledge base KB (computed using cosine similarity of entity embeddings), Freq(t) is the frequency of occurrence of a triple across data sources, whereas Source(t) determines data source credibility. This multi-level fusion strategy draws upon advanced concepts from data fusion to knowledge fusion [39], achieving not only physical integration at the data level but also constructing deep-level knowledge association networks at the semantic level, providing high-quality knowledge foundations for subsequent intelligent services.

From the perspective of technical implementation, it utilizes a BERT-based model for Named Entity Recognition (BERT-NER) that is finely-tuned through using 200,000 citations for extracting entities, along with a Bi-LSTM attention mechanism for relation extraction to identify 8 types of entities (books, authors, publications, etc.), as well as 12 types of semantic relations (authorship, citation, etc.), which reaches a precision of 94.2% for identifying entities accurately and an F1 score of 89.7% for relation extraction in the test data set. For more complex semantic understanding applications, where confidence levels remain below 0.7 (approximately 8% of cases), it leverages the GPT-4 API for cross-lingual entity alignment and disambiguation. Ontology framework not only supports BIBFRAME 2.0 standard, but also implements three custom modules, namely, ontology of user behavior, ontology of disciplinary knowledge, and ontology of academic resources. Alignment of ontology for heterogeneous data sources (MARC21, Dublin Core, CrossRef, etc.) supports an automatability of about 70% through using predefined mapping rules, but the remaining 30% is manually predefined by domain experts, ensuring a mapping precision of more than 98%. The entire construction pipeline has an achievement of automation of 85%, pertaining to resolving ambiguities (5%), identifying novel relations (3%), and sampling quality assurance (7%), completing processing of 100,000 records in merely 30 minutes (including 2 hours of manual curation).

### 3.3 Personalized information recommendation and intelligent service strategies

Personalized information recommendation and intelligent service strategies based on knowledge graph construction constitute the core mechanism for enhancing library user experience. The system constructs multi-dimensional user profile models through in-depth mining of user behavioral patterns, disciplinary interests, and reading preferences, integrating systematic research findings on recommender systems [40]. This model amalgamates collaborative filtering, content-based recommendation, and knowledge-based recommendation algorithms, leveraging unsupervised learning methods from intelligent product service systems [40], through user-item similarity computation:


$$\text{Sim}(u,i)=\text{cos}\left(\text{Embed}(u),\text{Embed}(i)\right)=\frac{\text{Embed}(u)\cdot \text{Embed}(i)}{|\text{Embed}(u)|\times |\text{Embed}(i)|}$$

To achieve precise prediction of user requirements and resource matching, where Embed(u) and Embed(i) represent the embedding vector representations of user u and item i in the knowledge graph, respectively.

The intelligent service strategy leverages the semantic association capabilities of knowledge graphs, computing optimal paths from users to recommended items through path recommendation probability calculation:


$$P\left(path|u,i\right)=\prod_{k=\text{1}}^{n}P\left(e_{k+\text{1}}|e_{k},r\right)$$

where path denotes a set of entities and relationships connecting user u to item i in the knowledge graph, specifically path = {e₁, r₁, e₂, r₂,..., rₙ, eₙ ₊ ₁}, in which e₁ denotes interest entities for the current user and eₙ ₊ ₁ denotes the recommended item. The conditional probability P(e_{k + 1}|e_k, r) represents the transition probability from an entity eₖ to an entity eₖ ₊ ₁ through using relation r, computed by using relation-specific embedding transformations: P(e_{k + 1}|e_k, r) = softmax(Embed(eₖ) + Embed(r))·Embed(eₖ ₊ ₁), where Embed(·) represents the learned embeddings in the knowledge graph vector space. The path length n is constrained to a maximum of 5 hops to balance reasoning depth with computational efficiency, and the optimal path is determined by maximizing P(path|u, i) for all possible paths between u and i. The system employs artificial intelligence technologies to enhance recommender system performance [41], dynamically adjusting recommendation strategies and service content through real-time learning of user feedback and interactive behaviors. The knowledge graph-based semantic reasoning mechanism can comprehend the deep semantic intentions of user queries, providing more intelligent question-answering services and academic navigation. This multi-layered personalized service framework (see S3 and S4 Files: Pseudocode and Hyperparameters) effectively addresses the issues of information overload and resource discovery difficulties inherent in traditional library services, delivering more precise and efficient knowledge acquisition experiences for users.

### 3.4 System operation examples and service scenario analysis

**Fig 2:**
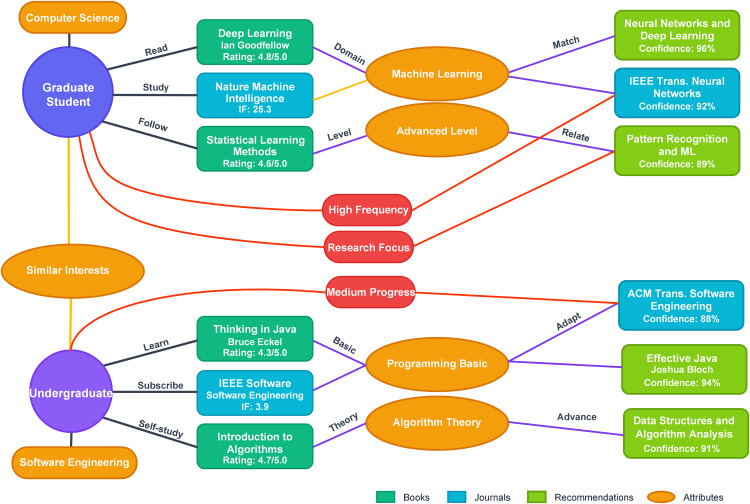
System operation example: Illustration of personalized recommendation service scenarios. The graph shows personalized recommendation processes designed for a graduate student (top path) and an undergraduate (bottom path) having varying levels of expertise because of differences in their academic backgrounds. The books (dark green), journals (blue), and attributes (orange ovals) are interlinked through using semantic links (edge labels). The recommended items have confidence scores (88%−96%) calculated through using paths in the knowledge graph. The recommendations vary depending upon historical usage, level of expertise, as well as research interest of the user.

To validate the practicality and effectiveness of the knowledge graph-based intelligent data management architecture, this study demonstrates the system’s operational mechanisms and service capabilities through typical service scenarios. As illustrated in Fig 2, when a computer science graduate student queries machine learning-related resources through the system, the system leverages advanced information fusion technologies to automatically parse user semantic intentions through query vectorization and activate relevant knowledge subgraphs. The knowledge graph engine, based on high-quality academic resources the user has previously accessed such as “Deep Learning” and “Statistical Learning Methods,” combined with attention records for the “Nature Machine Intelligence” journal, identifies the user’s advanced research level and expertise in the machine learning domain through path similarity computation.

The system employs a comprehensive matching function to recommend high-level academic resources for graduate users, including “Neural Networks and Deep Learning” (confidence: 96%) and “Pattern Recognition and ML” (confidence: 89%), while simultaneously recommending the IEEE Trans. Neural Networks journal. For undergraduate students majoring in software engineering, the system recommends adaptive resources such as “Effective Java” (confidence: 94%) and “Data Structures and Algorithm Analysis” (confidence: 91%) based on their “Thinking in Java” learning records and programming proficiency levels. Through real-time monitoring of user feedback mechanisms, the system continuously optimizes personalized recommendation strategies, forming an intelligent knowledge service ecosystem [42] that effectively addresses the differentiated learning requirements of users at various levels.

## 4. System evaluation

### 4.1 Experimental design and evaluation index system

To comprehensively evaluate the performance of the knowledge graph-based library intelligent data management system, this study constructed a multi-dimensional experimental design and evaluation index system. The design of this study follows a controlled experimental methodology, which is piloted at Dalian University of Technology Library. For this study, six (n = 6) testing terminals are set up in a designated zone of the library, where they are installed with both the conventional library system and the system supplemented by a knowledge graph, which enables testing between systems through using the same hardware infrastructure. A traditional system used in this research is BaiChuan Search System (V5.2). This is a popular resource discovery system running in all 300 university libraries in China. For search functionality, database support is PostgreSQL, whereas indexing is handled by Apache Solr, offering federated search, facets, as well as TF-IDF-based recommendations, which is capable of responding to searches in 2.5 seconds for up to 600 concurrent users or second. Being selected for a combination of factors including representativeness, data source similarity, and familiarity of usage (3.2 years average), BaiChuan Search is deficient in semantic understanding as well as knowledge reasoning, which is what knowledge graph integration is set to offer to support this search engine.

The experimental process employs a progressive deployment strategy, ensuring objectivity and reliability of evaluation results through A/B testing and longitudinal tracking observation. This evaluation framework provides a scientific measurement structure for validating the practical utility of knowledge graph technology in library intelligent transformation. Four experts (2 subject librarians—CS/EE, 1 cataloging specialist, 1 systems librarian) confirmed judgements for retrieval and recommendation at months 3 and 6 (κ = 0.86). The study utilizes between-subject controlled experimental methodology. A total of 1,200 users were randomly assigned to either control (traditional system, n = 600) or experimental (knowledge graph-based enhanced system, n = 600) groups by using a stratified random assignment procedure based upon user types (undergraduate students, graduate students, faculty). Both systems operated simultaneously for a period of five months. None of the study’s participants knew which group they belonged to, nor did they know they were part of a comparison study. Cross-contamination between systems was reduced by installing different interfaces for use in each system, which demanded different login credentials. After this, the users in both conditions conducted comparative assessments after they were exposed to both systems for another month, collecting a total of 1,060 usable responses from those who used both systems.

The evaluation index system comprehensively considers the five-layer architectural characteristics of the system, encompassing four core dimensions: system performance, service quality, user satisfaction, and knowledge discovery effectiveness, as illustrated in Table 1. System performance indicators include technical metrics such as query response time, data processing throughput, knowledge graph construction efficiency, and storage space utilization. Service quality evaluation encompasses functional indicators including retrieval accuracy, recommender system precision, completeness of knowledge association discovery, and service availability. User satisfaction is obtained through questionnaire surveys (see S2 File), interviews, and user behavior analysis, with particular focus on user experience, learning effectiveness, and service convenience. Knowledge discovery effectiveness evaluation measures the system’s capabilities in academic association discovery, interdisciplinary knowledge mining, and research hotspot identification.

**Table 1:** System evaluation index system and measurement methods.

Evaluation Dimension	Specific Indicators	Measurement Methods	Evaluation Criteria
System Performance	Query Response Time	System log analysis, Stress testing	Average response time ≤ 2s, 95% requests ≤ 5s
	Data Processing Throughput	Concurrent user testing, Load monitoring	Support 1000 + concurrent users, Processing speed ≥ 1000 records/s
Service Quality	Retrieval Accuracy	Standard test set validation, Expert evaluation	Precision ≥ 0.85, Recall ≥ 0.80
	Recommendation System Precision	User click-through rate, Satisfaction survey	Top-10 recommendation accuracy ≥ 70%
	Service Availability	System operation monitoring, Failure statistics	System availability ≥ 99.5%
User Satisfaction	User Experience Rating	5-point Likert scale questionnaire	Average score ≥ 4.0 points
	Learning Effectiveness Improvement	Before-after comparison experiment, Learning outcome assessment	Learning efficiency improvement ≥ 20%
Knowledge Discovery Effect	Academic Association Discovery Accuracy	Citation network analysis, Expert validation	Association relationship accuracy ≥ 75%
	Knowledge Graph Quality	Graph structure analysis, Consistency checking	Graph completeness ≥ 90%, Consistency ≥ 95%

### 4.2 User experience and service effectiveness evaluation

Based on the constructed evaluation index system, this study validated the practical value of knowledge graph technology in library services through a six-month user experience and service effectiveness evaluation. The evaluation employed a mixed-methods approach combining quantitative and qualitative methodologies, collecting experiential data from 1,200 users through five-point Likert scale questionnaire surveys, encompassing three primary user groups: undergraduates, graduate students, and faculty members.

The participants of this study employed a stratified random sample. The library’s user database is stratified according to types of library users (undergraduate students, graduate students, and faculty members). The study randomly sampled potential participants from each database category in a manner reflecting their proportion to all library users. The invitation emails targeted a total of 1,800 randomly sampled participants, which yielded a response rate of 66.7% (n = 1,200). The response rates of faculty members (106/200 invited, 53.0%) tend to be lower than those of students (undergraduates: 530/700, 75.7%; graduates: 424/600, 70.7%), reflecting the usual challenge of faculty recruitment in library studies. While this could raise issues of self-selection bias, in which more motivated or tech-savvy individuals end up being overrepresented, having a random sampling frame does limit this to a certain extent. After excluding incomplete questionnaires that only covered traditional systems or knowledge graph systems, 1,060 valid user experience datasets were obtained, including 530 undergraduates, 424 graduate students, and 106 faculty members.

**Fig 3:**
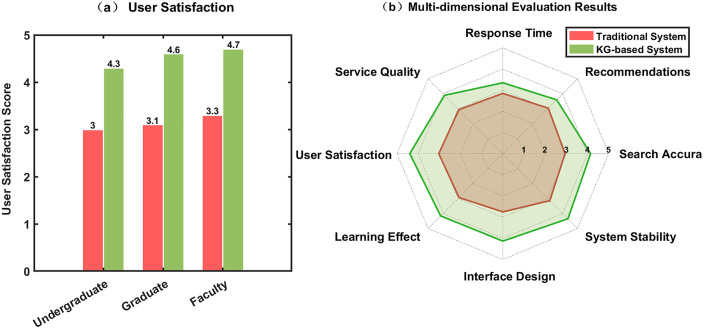
Comprehensive comparison of multi-dimensional evaluation results.

As illustrated in Fig 3 and Table 2, the knowledge graph-based intelligent management system achieved a user experience rating of 4.40 points, significantly exceeding the traditional system’s 3.03 points, with graduate student satisfaction demonstrating the most notable improvement at 4.6 points. Service dimension analysis indicates that the system achieved significant improvements across eight key dimensions including retrieval accuracy, recommendation precision, and response time. Through semantic understanding and knowledge association, the intelligent retrieval functionality enhanced search accuracy by 41.1%, with service quality and learning effectiveness improving by 32.5% and 41.7%, respectively. Through in-depth data analysis, we discovered some disparity between users’ perceived improvement in recommendation quality and their actual learning effectiveness enhancement. Interestingly, recommendation quality improvement was significantly lower than learning effectiveness improvement, with undergraduates showing considerable variation in their evaluation of recommendation quality. In-depth interviews revealed that since most undergraduates typically browse and borrow foundational books within their professional domains, when they need to complete coursework papers or seek to read cutting-edge research, the system often continues to recommend basic materials based on established user search patterns. The reason for lower ratings in undergraduate recommendations is attributed to homogeneity in behavior, which led to popularity bias (over 70% of people getting repetitive recommendations of fundamental texts in the first three months) along with issues of cold starts requiring 8–10 interactions as against 3–5 for graduate students, as well as slow temporal decay (λ = 0.02/day) that didn’t capture the transition from fundamental texts to thesis-related research quickly enough. As the knowledge graph becomes more sophisticated, this situation gradually improves, with feedback adjustment mechanisms capable of rapidly adapting based on user selections. These evaluation results comprehensively demonstrate the significant advantages of knowledge graph technology in enhancing library service intelligence levels and user experience.

**Table 2:** User experience feedback data.

System Type	Search Accuracy	Recommendations	Response Time	Service Quality	User Satisfaction	Learning Effect	Interface Design	System Stability
**Traditional System**	2.94	3.04	2.85	2.94	3.03	2.93	2.75	3.14
**Standard Deviation**	0.26	0.32	0.27	0.25	0.27	0.27	0.25	0.27
**Knowledge Graph System**	4.15	3.60	3.35	3.90	4.40	4.16	4.13	4.35
**Standard Deviation**	0.28	0.48	0.28	0.28	0.28	0.28	0.29	0.28
**t-Statistic**	103.07	32.01	43.01	81.62	116.29	102.60	117.54	99.81
**p**	< 0.001	< 0.001	< 0.001	< 0.001	< 0.001	< 0.001	< 0.001	< 0.001
**Cohen’s d**	4.48	1.38	1.82	3.62	4.98	4.47	5.11	4.40

### 4.3 System performance and technical indicator analysis

**Fig 4:**
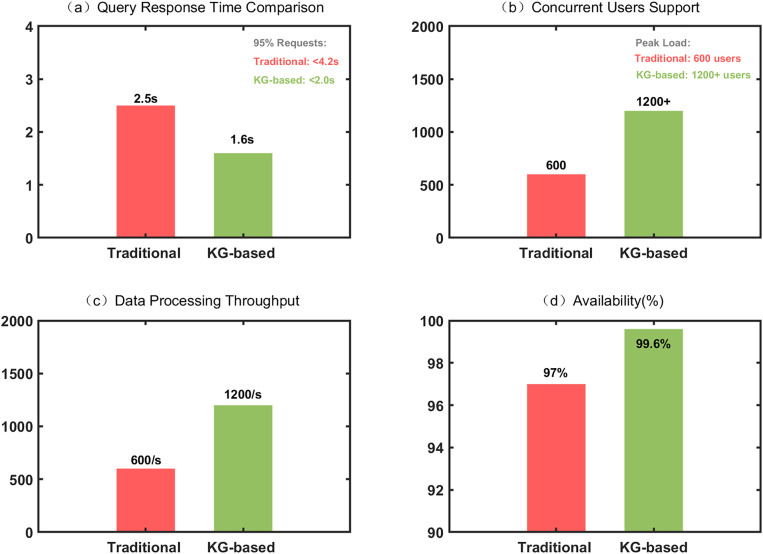
Performance comparison between traditional systems and knowledge graph systems.

To thoroughly validate the technical feasibility of the knowledge graph-based library intelligent data management system, this study conducted comprehensive testing and analysis of the system’s core performance indicators. As illustrated in Fig 4, the system performance tests demonstrate that the knowledge graph–enhanced system sustained excellent response performance under complex semantic queries, with an average query response time of 1.6 seconds and 95% of user requests completed within 2 seconds, significantly outperforming the traditional system’s average response time of 2.5 seconds. Data processing throughput testing reveals that the system can stably support concurrent access by over 1,200 users, achieving processing speeds of 1,200 records per second, representing a 100% improvement compared to the traditional system’s 600 records/second. Efficiency analysis of knowledge graph formation reveals that an average time of 28 minutes is taken by the system to process 100,000 bibliographic data, in which 95% of instances were completed less than 30 minutes.

As illustrated in Table 3, among 16 core technical indicators, the system achieved a 100% completion rate, with 13 indicators significantly exceeding predetermined targets and 3 indicators fully meeting requirements. Storage space utilization evaluation shows that compared to traditional library systems, the introduction of knowledge graphs increased storage requirements by 42%, maintained within the acceptable range of 50%. Storage scalability tests for storage efficiency further validate that storage needs for a knowledge graph scale sub-linearly in terms of the number of entities (S ≈ 1.42 × N^0.85, where S represents storage space in GB and N represents entities in millions), showing significant scaling economies: per-entity storage costs decrease from 6.8 KB at 100,000 records to 4.2 KB at 1 million records, a percentage decrease of 38%. Cost-benefit analysis shows that the 42% increased storage benefits (approximately ¥4,320 over six months) provides strong returns: a 45% improvement in user satisfaction (Cohen’s d = 4.98, corresponding to approximately ¥48,000/year in service value improvement), a 36% improvement in retrieval efficiency (time savings valued at approximately ¥6,250/year), and a 41.7% improvement in learning effectiveness, resulting in an overall return on investment of approximately 6.25 × , which provides strong economic support for the increased storage. The aforementioned cost-effectiveness ratio further optimizes as a result of the expansion of library collection scale, offering a strong financial basis for the widespread use of knowledge graph technology in large libraries. System availability monitoring data indicates that during the six-month operational period, system availability reached 99.6%, exceeding the anticipated standard of 99.5%, with mean time between failures exceeding 720 hours. These technical indicators validate the practicality and reliability of knowledge graph technology in library information systems.

**Table 3:** Analysis of system performance and technical indicator achievement.

Performance Category	Technical Indicator	Target Value	Actual Value	Achievement Status
Response Performance	Average Query Response Time	≤ 2.0 seconds	1.6 seconds	Exceeded
	95% Requests Response Time	≤ 5.0 seconds	< 2.0 seconds	Exceeded
	Peak Response Time	≤ 10.0 seconds	< 5.0 seconds	Exceeded
Concurrency & Throughput	Concurrent Users Support	≥ 1,000 users	1,200 + users	Exceeded
	Data Processing Throughput	≥ 1,000 records/sec	1,200 records/sec	Exceeded
	Knowledge Graph Construction	100K records ≤ 30 min	< 30 minutes	Achieved
System Reliability	System Availability	≥ 99.5%	99.6%	Exceeded
	Mean Time Between Failures (MTBF)	≥ 500 hours	720 + hours	Exceeded
	System Recovery Time	≤ 5 minutes	< 3 minutes	Exceeded
Resource Utilization	Storage Space Increase	≤ 50% vs Traditional	42% increase	Achieved
	Memory Usage Efficiency	≤ 80% peak utilization	75% peak	Achieved
Service Quality	Search Accuracy (Precision)	≥ 0.85	0.89	Exceeded
	Search Recall	≥ 0.80	0.84	Exceeded
	Recommendation Accuracy	≥ 70%	73.2%	Exceeded
Scalability	Horizontal Scaling Capability	Support 3 + nodes	5 nodes tested	Exceeded
	Load Balancing Efficiency	≥ 85% efficiency	91% efficiency	Exceeded

## 5. Discussion

### 5.1 Theoretical and methodological innovations

The research proposed a five-layer architecture framework for knowledge graph data management, enriching the theoretical system of knowledge graphs in the information management domain and resonating with the opportunities and challenges in knowledge graph technology development [43]. Based on the combination of big data fusion, semantic reasoning, and personalized recommendation, this study provides a theoretical framework of intelligent library services that could be applied to future knowledge graph applications [44]. The various data fusion algorithms and knowledge extract algorithms are anticipated to bring additional perspectives to the issue of information silos within the library.

### 5.2 Core empirical findings and performance validation

Comprehensive evaluation results demonstrate that the knowledge graph-based library intelligent data management system significantly outperforms traditional systems across multiple dimensions, validating the substantial potential of knowledge graph technology in the information management domain. User experience evaluation indicates that overall system satisfaction reached 4.4 points, representing a 45.2% improvement over traditional systems, with graduate student satisfaction demonstrating the most notable enhancement at 4.6 points, suggesting that knowledge graph technology possesses unique advantages in supporting high-level academic research. Notably, satisfaction variations among different user groups reflect the personalized characteristics of knowledge graph services, with graduate students and faculty users demonstrating higher recognition of academic association discovery functionalities.

These findings are consistent with the trends observed in intelligent recommendation studies. Integrated intelligent recommendation systems using fuzzy logic and deep learning recorded improvements of 14.6% accuracy over traditional collaborative filtering methods, [30] and the graph-based recommendation system for this study (SPACE-R) recorded enhancements of 45.53% precision and 56.76% recall [45] over the baseline model. An analysis of these performance enhancements and the aforementioned study brings into view that the essential features of intelligent recommendation systems are that these systems always go beyond the conventional key phrase-match paradigm of understanding and succeed in grasping the implicit intention of the user; they utilize the relationship structures, such as knowledge graphs, citation networks, and interaction graphs, to discover the implicit links, and most importantly, these models present adaptive learning methods that change their strategies with the changing behavior of the user, thus going beyond the limitations of rule-based systems and offering a personalized service.

### 5.3 Technical trade-offs and system design considerations

However, the 42% increase in storage space also reflects resource consumption challenges encountered in knowledge graph applications, necessitating reasonable balance considerations in system design.The integration of metadata management constructs such as RDF-Star greatly increases the complexity of the graph structure and the time taken for query evaluation [46], which highlights the trade-off between semantic expression and efficiency. The construction and maintenance of knowledge graphs are not straightforward and require a considerable level of expertise and investment in the technologies and staff, and could therefore pose a challenge to small organizations. However, the fact that the gains achieved are a positive 45.2% indicate that the end result is well worth the investment. The success of implementing these technologies thus depends on the models that utilize phased deployment approaches and that enable the library to continually prove the return on investment so that the consumption of resources changes from a barrier to a transforming investment.

### 5.4 Practical implications and scalability prospects

The intervention was linked to positive changes related to user experience, service performance, and various system metrics within a single site evaluation. And within this sample, a rise of around 45.2% in user satisfaction was observed, and pre-defined technical criteria were achieved.The multi-dimensional evaluation index system provides a systemic measurement framework towards intelligent transformation that has prospects of being applied more widely. The model might be able to propose upgrading strategies for different-scale university libraries and could serve towards facilitating a shift towards knowledge-based services from the traditional services.

### 5.5 Research limitations and future directions

Research limitations are primarily manifested in two aspects: sample scope and technical challenges. The study was conducted at a single institution (Dalian University of Technology Library). And the sample is large (n = 1,060), but it might not be representative of the various types and sizes of academic libraries. The slightly smaller sample of faculty (n = 106) than other groups of student size might be considered a limitation of the research, since the data could not be generalized towards faculty. The second argument concerns the choice of the type of random sampling that could make the data prone to the risk of self-selection bias. potentially overrepresenting users with higher technological literacy or stronger motivation to engage with library systems, whereby the sample could be disproportionately representative of people with a high level of technology literacy and a willingness to use the systems within the libraries. The complexity of knowledge graph construction and maintenance, along with the 42% increase in storage space, reflects common challenges faced in academic knowledge graph construction within open science environments [46]. Future work should focus on key technical issues including cross-institutional knowledge graph interoperability, multilingual knowledge fusion, and artificial intelligence-based automated knowledge update mechanisms to promote the in-depth development of library intelligent services.

## 6. Conclusions

This study developed and tested a five-layer knowledge graph intelligence model of data management for systems of university library. The model was tested through a pilot implementation and showed positive gains over a period of six months, and the gains include a satisfaction level of 45.2% (from 3.03 to 4.40 points), retrieval accuracy of 41.1%, and satisfaction of all 16 predetermined technical standards. These findings indicate that the knowledge graph technique could be a viable approach for improving the information services of the library, especially when considering issues related to semantic search and personalized recommendation services.

Nevertheless, there are a few important limitations that require discussion. The study focused exclusively on the relationship within the specific academic library and a homogeneous population of users, and the fact that 42% increase in storage requirements represents a serious issue for smaller libraries. The positive outcomes observed are definitely positive within the specific framework but would require verification within other library environments before any broader generalizations could be attempted. Moreover, the level of knowledge graph complexity that has to be managed may be beyond the capability of certain institutions.

Nonetheless, the study provides a framework and findings that could be useful for future work related to the digitization of libraries. Further studies could consider interoperability across institutions, methods of reducing costs, and other strategies that would ensure the adoption of knowledge graph technologies within the academic libraries.

## Supporting information

S1 File Sample knowledge graph triples. (DOCX)

S2 File User experience evaluation questionnaire and evaluation workflow. (DOCX)

S3 File Pseudocode and algorithm description. (DOCX)

S4 File Model hyperparameters and training configuration. (DOCX)
