# Supplementary material for: Knowledge graph-based intelligent data management and information innovation service model for university library systems
Source: PLoS One. 2026 Jan 16;21(1):e0341307. doi: 10.1371/journal.pone.0341307 (PMC12810841; doi:10.1371/journal.pone.0341307)
Supplement: S1 File — (DOCX) [file pone.0341307.s001.docx]

Supplementary File S1: Sample Knowledge Graph Triples

{

"knowledge_graph_sample": {

"description": "Representative sample of knowledge graph triples from the university library system. For privacy protection, all user IDs are anonymized and sensitive personal information is removed.",

"statistics": {

"total_entities_in_full_kg": 2530000,

"total_relations_in_full_kg": 4010000,

"sample_size": 100,

"entity_types": 8,

"relation_types": 12

},

"triples": [

{

"id": "T001",

"subject": "Book_001",

"subject_type": "Book",

"subject_label": "Deep Learning",

"predicate": "authored_by",

"object": "Author_001",

"object_type": "Author",

"object_label": "Ian Goodfellow",

"confidence": 0.98

},

{

"id": "T002",

"subject": "Book_001",

"subject_type": "Book",

"subject_label": "Deep Learning",

"predicate": "published_by",

"object": "Publisher_001",

"object_type": "Publisher",

"object_label": "MIT Press",

"confidence": 0.99

},

{

"id": "T003",

"subject": "Book_001",

"subject_type": "Book",

"subject_label": "Deep Learning",

"predicate": "belongs_to_discipline",

"object": "Discipline_001",

"object_type": "Discipline",

"object_label": "Computer Science",

"confidence": 0.97

},

{

"id": "T004",

"subject": "Book_001",

"subject_type": "Book",

"subject_label": "Deep Learning",

"predicate": "has_keyword",

"object": "Keyword_001",

"object_type": "Keyword",

"object_label": "Neural Networks",

"confidence": 0.95

},

{

"id": "T005",

"subject": "User_A001",

"subject_type": "User",

"subject_label": "Anonymous_Graduate_Student_001",

"predicate": "borrowed",

"object": "Book_001",

"object_type": "Book",

"object_label": "Deep Learning",

"confidence": 1.0,

"timestamp": "2024-03-15"

},

{

"id": "T006",

"subject": "User_A001",

"subject_type": "User",

"subject_label": "Anonymous_Graduate_Student_001",

"predicate": "interested_in",

"object": "Discipline_001",

"object_type": "Discipline",

"object_label": "Computer Science",

"confidence": 0.92

},

{

"id": "T007",

"subject": "Book_002",

"subject_type": "Book",

"subject_label": "Pattern Recognition and Machine Learning",

"predicate": "authored_by",

"object": "Author_002",

"object_type": "Author",

"object_label": "Christopher Bishop",

"confidence": 0.99

},

{

"id": "T008",

"subject": "Book_001",

"subject_type": "Book",

"subject_label": "Deep Learning",

"predicate": "cited_by",

"object": "Article_001",

"object_type": "Article",

"object_label": "Attention Is All You Need",

"confidence": 0.96

},

{

"id": "T009",

"subject": "Book_001",

"subject_type": "Book",

"subject_label": "Deep Learning",

"predicate": "similar_to",

"object": "Book_002",

"object_type": "Book",

"object_label": "Pattern Recognition and Machine Learning",

"confidence": 0.88

},

{

"id": "T010",

"subject": "Author_001",

"subject_type": "Author",

"subject_label": "Ian Goodfellow",

"predicate": "affiliated_with",

"object": "Institution_001",

"object_type": "Institution",

"object_label": "Google Brain",

"confidence": 0.94

},

{

"id": "T011",

"subject": "Journal_001",

"subject_type": "Journal",

"subject_label": "Nature Machine Intelligence",

"predicate": "belongs_to_discipline",

"object": "Discipline_002",

"object_type": "Discipline",

"object_label": "Artificial Intelligence",

"confidence": 0.98

},

{

"id": "T012",

"subject": "User_A001",

"subject_type": "User",

"subject_label": "Anonymous_Graduate_Student_001",

"predicate": "accessed",

"object": "Journal_001",

"object_type": "Journal",

"object_label": "Nature Machine Intelligence",

"confidence": 1.0,

"timestamp": "2024-04-10"

},

{

"id": "T013",

"subject": "Book_003",

"subject_type": "Book",

"subject_label": "Effective Java",

"predicate": "authored_by",

"object": "Author_003",

"object_type": "Author",

"object_label": "Joshua Bloch",

"confidence": 0.99

},

{

"id": "T014",

"subject": "Book_003",

"subject_type": "Book",

"subject_label": "Effective Java",

"predicate": "belongs_to_discipline",

"object": "Discipline_003",

"object_type": "Discipline",

"object_label": "Software Engineering",

"confidence": 0.96

},

{

"id": "T015",

"subject": "User_B001",

"subject_type": "User",

"subject_label": "Anonymous_Undergraduate_001",

"predicate": "borrowed",

"object": "Book_003",

"object_type": "Book",

"object_label": "Effective Java",

"confidence": 1.0,

"timestamp": "2024-05-20"

},

{

"id": "T016",

"subject": "Book_004",

"subject_type": "Book",

"subject_label": "Thinking in Java",

"predicate": "authored_by",

"object": "Author_004",

"object_type": "Author",

"object_label": "Bruce Eckel",

"confidence": 0.98

},

{

"id": "T017",

"subject": "Book_003",

"subject_type": "Book",

"subject_label": "Effective Java",

"predicate": "references",

"object": "Book_004",

"object_type": "Book",

"object_label": "Thinking in Java",

"confidence": 0.87

},

{

"id": "T018",

"subject": "User_B001",

"subject_type": "User",

"subject_label": "Anonymous_Undergraduate_001",

"predicate": "interested_in",

"object": "Discipline_003",

"object_type": "Discipline",

"object_label": "Software Engineering",

"confidence": 0.89

},

{

"id": "T019",

"subject": "Discipline_001",

"subject_type": "Discipline",

"subject_label": "Computer Science",

"predicate": "interdisciplinary_with",

"object": "Discipline_002",

"object_type": "Discipline",

"object_label": "Artificial Intelligence",

"confidence": 0.93

},

{

"id": "T020",

"subject": "Conference_001",

"subject_type": "Conference",

"subject_label": "NeurIPS 2024",

"predicate": "belongs_to_discipline",

"object": "Discipline_002",

"object_type": "Discipline",

"object_label": "Artificial Intelligence",

"confidence": 0.97

}

],

"entity_type_definitions": {

"Book": "Bibliographic records of books in the library collection",

"Author": "Authors of books, articles, and other publications",

"Publisher": "Publishing houses and academic presses",

"Discipline": "Academic disciplines and subject areas",

"Keyword": "Subject keywords and descriptors",

"User": "Library users (anonymized identifiers)",

"Journal": "Academic journals and periodicals",

"Conference": "Academic conferences and proceedings"

},

"relation_type_definitions": {

"authored_by": "Book/Article is written by Author",

"published_by": "Book is published by Publisher",

"belongs_to_discipline": "Resource belongs to academic Discipline",

"has_keyword": "Resource is tagged with Keyword",

"borrowed": "User borrows Book",

"interested_in": "User shows interest in Discipline/Topic",

"cited_by": "Resource is cited by another Resource",

"similar_to": "Resources are semantically similar",

"affiliated_with": "Author is affiliated with Institution",

"accessed": "User accessed Journal/Resource",

"references": "Resource references another Resource",

"interdisciplinary_with": "Discipline has interdisciplinary connection"

}

}

}
