# Supplementary material for: Knowledge graph-based intelligent data management and information innovation service model for university library systems
Source: PLoS One. 2026 Jan 16;21(1):e0341307. doi: 10.1371/journal.pone.0341307 (PMC12810841; doi:10.1371/journal.pone.0341307)
Supplement: S2 File — (DOCX) [file pone.0341307.s002.docx]

Supplementary File S2: User Experience Evaluation Questionnaire and evaluation workflow

**Initial recruitment**: 1,800 users invited via stratified random sampling

**Consented participants**: 1,200 users (66.7% participation rate)

**Questionnaire surveys**: All 1,200 participants

**Behavioral data collection**: All 1,200 participants (automated system logs)

**Semi-structured interviews**: Purposive subsample of 60 participants (30 from each system group, selected to represent diverse satisfaction levels and user types: 30 undergraduates, 20 graduates, 10 faculty)

**Complete data**: 1,060 participants with valid questionnaires + behavioral logs

**Interview completions**: 58/60 (96.7% completion rate)

**Interview Structure:** Semi-structured interviews (n=60, duration 15-20 minutes) were conducted with a purposive subsample at Month 5. Interview protocol included: (1) System usage patterns and frequency, (2) Perceived strengths and weaknesses of each system, (3) Specific examples of successful/unsuccessful information retrieval, (4) Suggestions for improvement. Interviews were audio-recorded with consent, transcribed verbatim, and analyzed using thematic analysis to identify recurring patterns. The finding regarding undergraduate recommendation quality issues emerged from this qualitative analysis, corroborated by quantitative survey data.

Data synthesis followed a convergent mixed-methods design: (1) Quantitative data (questionnaires + behavioral logs) were analyzed using paired t-tests and descriptive statistics to identify performance differences, (2) Qualitative data (interview transcripts) were thematically coded to explain quantitative patterns and uncover user perceptions, (3) Integration occurred through triangulation—comparing quantitative trends with qualitative themes to validate findings and provide explanatory depth

# User Experience Evaluation Questionnaire

## Knowledge Graph-Based Library Intelligent Management System

Research Purpose: This questionnaire evaluates your experience with both the traditional library system and the knowledge graph-enhanced system across multiple dimensions. Your honest feedback is crucial for assessing system effectiveness.

Estimated Time: 15-20 minutes

Confidentiality: All responses are anonymous and will be used solely for research purposes. Your participation is voluntary.

---

## Part A: Participant Information

1. User Type:

☐ Undergraduate Student ☐ Graduate Student (Master's) ☐ Graduate Student (PhD) ☐ Faculty Member

2. Major/Department: _______________________

3. Years of library system usage:

☐ Less than 1 year ☐ 1-2 years ☐ 3-4 years ☐ More than 5 years

4. Frequency of library system usage:

☐ Daily ☐ 3-5 times per week ☐ 1-2 times per week ☐ 2-3 times per month ☐ Less than once per month

5. Primary purpose of using the library system (select all that apply):

☐ Course readings ☐ Research ☐ Paper writing ☐ General learning ☐ Leisure reading

---

## Part B: Comparative Evaluation

Instructions: For each question below, please rate both systems using the following scale:

- 1 = Strongly Disagree / Very Poor

- 2 = Disagree / Poor

- 3 = Neutral

- 4 = Agree / Good

- 5 = Strongly Agree / Excellent

---

### Section 1: Search Accuracy

1.1 Relevance of Search Results

Traditional System: The search results matched my information needs accurately.

☐ 1 ☐ 2 ☐ 3 ☐ 4 ☐ 5

Knowledge Graph System: The search results matched my information needs accurately.

☐ 1 ☐ 2 ☐ 3 ☐ 4 ☐ 5

---

1.2 Ranking Quality

Traditional System: The most relevant resources appeared at the top of search results.

☐ 1 ☐ 2 ☐ 3 ☐ 4 ☐ 5

Knowledge Graph System: The most relevant resources appeared at the top of search results.

☐ 1 ☐ 2 ☐ 3 ☐ 4 ☐ 5

---

1.3 Understanding of Query Intent

Traditional System: The system correctly understood my search queries (including complex or ambiguous queries).

☐ 1 ☐ 2 ☐ 3 ☐ 4 ☐ 5

Knowledge Graph System: The system correctly understood my search queries (including complex or ambiguous queries).

☐ 1 ☐ 2 ☐ 3 ☐ 4 ☐ 5

---

1.4 Precision in Filtering

Traditional System: The system effectively filtered out irrelevant results.

☐ 1 ☐ 2 ☐ 3 ☐ 4 ☐ 5

Knowledge Graph System: The system effectively filtered out irrelevant results.

☐ 1 ☐ 2 ☐ 3 ☐ 4 ☐ 5

---

1.5 Overall Search Accuracy

Traditional System: Overall, how would you rate the search accuracy?

☐ 1 (Very Poor) ☐ 2 (Poor) ☐ 3 (Neutral) ☐ 4 (Good) ☐ 5 (Excellent)

Knowledge Graph System: Overall, how would you rate the search accuracy?

☐ 1 (Very Poor) ☐ 2 (Poor) ☐ 3 (Neutral) ☐ 4 (Good) ☐ 5 (Excellent)

Comments on Search Accuracy:

---

### Section 2: Recommendation Quality (推荐质量)

2.1 Relevance of Recommendations

Traditional System: The recommended resources were relevant to my interests and needs.

☐ 1 ☐ 2 ☐ 3 ☐ 4 ☐ 5

Knowledge Graph System: The recommended resources were relevant to my interests and needs.

☐ 1 ☐ 2 ☐ 3 ☐ 4 ☐ 5

---

2.2 Diversity of Recommendations

Traditional System: The system recommended diverse resources beyond my usual search patterns.

☐ 1 ☐ 2 ☐ 3 ☐ 4 ☐ 5

Knowledge Graph System: The system recommended diverse resources beyond my usual search patterns.

☐ 1 ☐ 2 ☐ 3 ☐ 4 ☐ 5

---

2.3 Timeliness of Recommendations

Traditional System: The recommendations included up-to-date resources appropriate to current research trends.

☐ 1 ☐ 2 ☐ 3 ☐ 4 ☐ 5

Knowledge Graph System: The recommendations included up-to-date resources appropriate to current research trends.

☐ 1 ☐ 2 ☐ 3 ☐ 4 ☐ 5

---

2.4 Discovery of New Resources

Traditional System: The system helped me discover valuable resources I wouldn't have found otherwise.

☐ 1 ☐ 2 ☐ 3 ☐ 4 ☐ 5

Knowledge Graph System: The system helped me discover valuable resources I wouldn't have found otherwise.

☐ 1 ☐ 2 ☐ 3 ☐ 4 ☐ 5

---

2.5 Overall Recommendation Quality

Traditional System: Overall, how would you rate the recommendation quality?

☐ 1 (Very Poor) ☐ 2 (Poor) ☐ 3 (Neutral) ☐ 4 (Good) ☐ 5 (Excellent)

Knowledge Graph System: Overall, how would you rate the recommendation quality?

☐ 1 (Very Poor) ☐ 2 (Poor) ☐ 3 (Neutral) ☐ 4 (Good) ☐ 5 (Excellent)

Comments on Recommendation Quality:

---

### Section 3: Response Time (响应时间)

3.1 Search Response Speed

Traditional System: The system returned search results quickly without noticeable delays.

☐ 1 ☐ 2 ☐ 3 ☐ 4 ☐ 5

Knowledge Graph System: The system returned search results quickly without noticeable delays.

☐ 1 ☐ 2 ☐ 3 ☐ 4 ☐ 5

---

3.2 Page Loading Speed

Traditional System: Pages and resources loaded quickly when browsing.

☐ 1 ☐ 2 ☐ 3 ☐ 4 ☐ 5

Knowledge Graph System: Pages and resources loaded quickly when browsing.

☐ 1 ☐ 2 ☐ 3 ☐ 4 ☐ 5

---

3.3 System Responsiveness During Peak Hours

Traditional System: The system maintained good performance even during busy periods (e.g., exam seasons).

☐ 1 ☐ 2 ☐ 3 ☐ 4 ☐ 5

Knowledge Graph System: The system maintained good performance even during busy periods (e.g., exam seasons).

☐ 1 ☐ 2 ☐ 3 ☐ 4 ☐ 5

---

3.4 Overall Response Time

Traditional System: Overall, how would you rate the system's response speed?

☐ 1 (Very Poor) ☐ 2 (Poor) ☐ 3 (Neutral) ☐ 4 (Good) ☐ 5 (Excellent)

Knowledge Graph System: Overall, how would you rate the system's response speed?

☐ 1 (Very Poor) ☐ 2 (Poor) ☐ 3 (Neutral) ☐ 4 (Good) ☐ 5 (Excellent)

Comments on Response Time:

---

### Section 4: Service Quality (服务质量)

4.1 Completeness of Features

Traditional System: The system provided all the features and functions I needed for my library tasks.

☐ 1 ☐ 2 ☐ 3 ☐ 4 ☐ 5

Knowledge Graph System: The system provided all the features and functions I needed for my library tasks.

☐ 1 ☐ 2 ☐ 3 ☐ 4 ☐ 5

---

4.2 Information Accuracy

Traditional System: The bibliographic information (author, title, publication date, etc.) was accurate and complete.

☐ 1 ☐ 2 ☐ 3 ☐ 4 ☐ 5

Knowledge Graph System: The bibliographic information (author, title, publication date, etc.) was accurate and complete.

☐ 1 ☐ 2 ☐ 3 ☐ 4 ☐ 5

---

4.3 Ease of Use

Traditional System: The system was intuitive and easy to use without extensive training.

☐ 1 ☐ 2 ☐ 3 ☐ 4 ☐ 5

Knowledge Graph System: The system was intuitive and easy to use without extensive training.

☐ 1 ☐ 2 ☐ 3 ☐ 4 ☐ 5

---

4.4 Advanced Search Capabilities

Traditional System: The system offered effective advanced search options (filters, Boolean operators, field-specific search).

☐ 1 ☐ 2 ☐ 3 ☐ 4 ☐ 5

Knowledge Graph System: The system offered effective advanced search options (filters, Boolean operators, field-specific search).

☐ 1 ☐ 2 ☐ 3 ☐ 4 ☐ 5

---

4.5 Overall Service Quality

Traditional System: Overall, how would you rate the service quality?

☐ 1 (Very Poor) ☐ 2 (Poor) ☐ 3 (Neutral) ☐ 4 (Good) ☐ 5 (Excellent)

Knowledge Graph System: Overall, how would you rate the service quality?

☐ 1 (Very Poor) ☐ 2 (Poor) ☐ 3 (Neutral) ☐ 4 (Good) ☐ 5 (Excellent)

Comments on Service Quality:

---

### Section 5: User Satisfaction (用户满意度)

5.1 Meeting Expectations

Traditional System: The system met my expectations for library services.

☐ 1 ☐ 2 ☐ 3 ☐ 4 ☐ 5

Knowledge Graph System: The system met my expectations for library services.

☐ 1 ☐ 2 ☐ 3 ☐ 4 ☐ 5

---

5.2 Likelihood to Recommend

Traditional System: I would recommend this system to other students/faculty.

☐ 1 ☐ 2 ☐ 3 ☐ 4 ☐ 5

Knowledge Graph System: I would recommend this system to other students/faculty.

☐ 1 ☐ 2 ☐ 3 ☐ 4 ☐ 5

---

5.3 Continued Use Intent

Traditional System: I would prefer to continue using this system for my library needs.

☐ 1 ☐ 2 ☐ 3 ☐ 4 ☐ 5

Knowledge Graph System: I would prefer to continue using this system for my library needs.

☐ 1 ☐ 2 ☐ 3 ☐ 4 ☐ 5

---

5.4 Time-Saving Effectiveness

Traditional System: The system saved me significant time in finding the resources I needed.

☐ 1 ☐ 2 ☐ 3 ☐ 4 ☐ 5

Knowledge Graph System: The system saved me significant time in finding the resources I needed.

☐ 1 ☐ 2 ☐ 3 ☐ 4 ☐ 5

---

5.5 Overall User Satisfaction

Traditional System: Overall, how satisfied are you with this system?

☐ 1 (Very Dissatisfied) ☐ 2 (Dissatisfied) ☐ 3 (Neutral) ☐ 4 (Satisfied) ☐ 5 (Very Satisfied)

Knowledge Graph System: Overall, how satisfied are you with this system?

☐ 1 (Very Dissatisfied) ☐ 2 (Dissatisfied) ☐ 3 (Neutral) ☐ 4 (Satisfied) ☐ 5 (Very Satisfied)

Comments on User Satisfaction:

---

### Section 6: Learning Effect (学习效果)

6.1 Support for Academic Work

Traditional System: The system effectively supported my coursework, research, or teaching activities.

☐ 1 ☐ 2 ☐ 3 ☐ 4 ☐ 5

Knowledge Graph System: The system effectively supported my coursework, research, or teaching activities.

☐ 1 ☐ 2 ☐ 3 ☐ 4 ☐ 5

---

6.2 Knowledge Discovery

Traditional System: The system helped me discover new knowledge and connections between topics.

☐ 1 ☐ 2 ☐ 3 ☐ 4 ☐ 5

Knowledge Graph System: The system helped me discover new knowledge and connections between topics.

☐ 1 ☐ 2 ☐ 3 ☐ 4 ☐ 5

---

6.3 Research Efficiency

Traditional System: The system improved my efficiency in conducting literature reviews and research.

☐ 1 ☐ 2 ☐ 3 ☐ 4 ☐ 5

Knowledge Graph System: The system improved my efficiency in conducting literature reviews and research.

☐ 1 ☐ 2 ☐ 3 ☐ 4 ☐ 5

---

6.4 Depth of Learning

Traditional System: The system facilitated deeper understanding of my subject area.

☐ 1 ☐ 2 ☐ 3 ☐ 4 ☐ 5

Knowledge Graph System: The system facilitated deeper understanding of my subject area.

☐ 1 ☐ 2 ☐ 3 ☐ 4 ☐ 5

---

6.5 Overall Learning Effect

Traditional System: Overall, how would you rate the system's contribution to your learning?

☐ 1 (Very Poor) ☐ 2 (Poor) ☐ 3 (Neutral) ☐ 4 (Good) ☐ 5 (Excellent)

Knowledge Graph System: Overall, how would you rate the system's contribution to your learning?

☐ 1 (Very Poor) ☐ 2 (Poor) ☐ 3 (Neutral) ☐ 4 (Good) ☐ 5 (Excellent)

Comments on Learning Effect:

---

### Section 7: Interface Design (界面设计)

7.1 Visual Appeal

Traditional System: The interface was visually appealing and professional.

☐ 1 ☐ 2 ☐ 3 ☐ 4 ☐ 5

Knowledge Graph System: The interface was visually appealing and professional.

☐ 1 ☐ 2 ☐ 3 ☐ 4 ☐ 5

---

7.2 Layout and Organization

Traditional System: The layout was well-organized and information was easy to locate.

☐ 1 ☐ 2 ☐ 3 ☐ 4 ☐ 5

Knowledge Graph System: The layout was well-organized and information was easy to locate.

☐ 1 ☐ 2 ☐ 3 ☐ 4 ☐ 5

---

7.3 Navigation Ease

Traditional System: Navigating between different sections and features was intuitive.

☐ 1 ☐ 2 ☐ 3 ☐ 4 ☐ 5

Knowledge Graph System: Navigating between different sections and features was intuitive.

☐ 1 ☐ 2 ☐ 3 ☐ 4 ☐ 5

---

7.4 Mobile Responsiveness (if applicable)

Traditional System: The interface worked well on mobile devices.

☐ 1 ☐ 2 ☐ 3 ☐ 4 ☐ 5 ☐ N/A (Did not use on mobile)

Knowledge Graph System: The interface worked well on mobile devices.

☐ 1 ☐ 2 ☐ 3 ☐ 4 ☐ 5 ☐ N/A (Did not use on mobile)

---

7.5 Overall Interface Design

Traditional System: Overall, how would you rate the interface design?

☐ 1 (Very Poor) ☐ 2 (Poor) ☐ 3 (Neutral) ☐ 4 (Good) ☐ 5 (Excellent)

Knowledge Graph System: Overall, how would you rate the interface design?

☐ 1 (Very Poor) ☐ 2 (Poor) ☐ 3 (Neutral) ☐ 4 (Good) ☐ 5 (Excellent)

Comments on Interface Design:

---

### Section 8: System Stability (系统稳定性)

8.1 Frequency of Errors

Traditional System: The system rarely experienced errors or crashes during my use.

☐ 1 ☐ 2 ☐ 3 ☐ 4 ☐ 5

Knowledge Graph System: The system rarely experienced errors or crashes during my use.

☐ 1 ☐ 2 ☐ 3 ☐ 4 ☐ 5

---

8.2 System Availability

Traditional System: The system was available whenever I needed to use it (minimal downtime).

☐ 1 ☐ 2 ☐ 3 ☐ 4 ☐ 5

Knowledge Graph System: The system was available whenever I needed to use it (minimal downtime).

☐ 1 ☐ 2 ☐ 3 ☐ 4 ☐ 5

---

8.3 Data Integrity

Traditional System: My saved searches, bookmarks, and borrowing records were reliably preserved.

☐ 1 ☐ 2 ☐ 3 ☐ 4 ☐ 5

Knowledge Graph System: My saved searches, bookmarks, and borrowing records were reliably preserved.

☐ 1 ☐ 2 ☐ 3 ☐ 4 ☐ 5

---

8.4 Error Recovery

Traditional System: When errors occurred, the system recovered gracefully without data loss.

☐ 1 ☐ 2 ☐ 3 ☐ 4 ☐ 5

Knowledge Graph System: When errors occurred, the system recovered gracefully without data loss.

☐ 1 ☐ 2 ☐ 3 ☐ 4 ☐ 5

---

8.5 Overall System Stability

Traditional System: Overall, how would you rate the system's stability and reliability?

☐ 1 (Very Poor) ☐ 2 (Poor) ☐ 3 (Neutral) ☐ 4 (Good) ☐ 5 (Excellent)

Knowledge Graph System: Overall, how would you rate the system's stability and reliability?

☐ 1 (Very Poor) ☐ 2 (Poor) ☐ 3 (Neutral) ☐ 4 (Good) ☐ 5 (Excellent)

Comments on System Stability:

---

## Part C: Open-Ended Questions

C1. What specific features or capabilities of the knowledge graph system did you find most valuable?

_________________________________________________________________

_________________________________________________________________

_________________________________________________________________

C2. What aspects of the knowledge graph system need improvement?

_________________________________________________________________

_________________________________________________________________

_________________________________________________________________

C3. Can you provide a specific example where the knowledge graph system helped you find resources more effectively than the traditional system?

_________________________________________________________________

_________________________________________________________________

_________________________________________________________________

C4. Were there any situations where the traditional system worked better than the knowledge graph system? Please describe.

_________________________________________________________________

_________________________________________________________________

_________________________________________________________________

C5. Additional comments or suggestions:

_________________________________________________________________

_________________________________________________________________

_________________________________________________________________

---

## Thank You!

Your participation in this evaluation is greatly appreciated. Your feedback will directly contribute to improving library services.

Estimated completion time: ______ minutes

Date completed: ________________
