# Supplementary material for: Knowledge graph-based intelligent data management and information innovation service model for university library systems
Source: PLoS One. 2026 Jan 16;21(1):e0341307. doi: 10.1371/journal.pone.0341307 (PMC12810841; doi:10.1371/journal.pone.0341307)
Supplement: S3 File — (DOCX) [file pone.0341307.s003.docx]

Supplementary File S3: Pseudocode and Algorithm Description

**Algorithm 1: Entity Recognition and Relation Extraction**

# Algorithm 1: Entity Recognition and Relation Extraction Pipeline

FUNCTION EntityRecognitionAndRelationExtraction(bibliographic_records):

INPUT: bibliographic_records - List of raw bibliographic records

OUTPUT: knowledge_triples - Set of (subject, predicate, object) triples

# Step 1: Preprocessing

cleaned_records = []

FOR EACH record IN bibliographic_records:

# Remove duplicates based on ISBN/DOI

IF NOT is_duplicate(record):

# Normalize encoding to UTF-8

normalized = normalize_encoding(record)

# Parse fields based on format (MARC21/Dublin Core/JSON)

parsed = parse_fields(normalized)

cleaned_records.APPEND(parsed)

# Step 2: Entity Recognition using BERT-NER

entities = []

FOR EACH record IN cleaned_records:

text = concatenate_fields(record, ['title', 'author', 'abstract'])

# Tokenize and encode for BERT

input_ids = bert_tokenizer.encode(text, max_length=512)

# BERT-NER inference

entity_predictions = bert_ner_model.predict(input_ids)

# Extract entities with confidence scores

FOR EACH token, label, score IN entity_predictions:

IF score >= CONFIDENCE_THRESHOLD: # 0.7

entity = {

'text': token,

'type': label, # Book, Author, Publisher, etc.

'confidence': score,

'source_record': record.id

}

entities.APPEND(entity)

ELSE:

# Low confidence: use GPT-4 for disambiguation

entity = gpt4_entity_disambiguation(token, context=text)

entities.APPEND(entity)

# Step 3: Relation Extraction using Bi-LSTM with Attention

relations = []

FOR EACH entity_pair IN get_candidate_pairs(entities):

subject, object = entity_pair

context = extract_context(subject, object, window_size=50)

# Encode context using Bi-LSTM

context_embedding = bilstm_model.encode(context)

# Apply attention mechanism

attention_weights = attention_layer(context_embedding)

attended_features = attention_weights context_embedding

# Classify relation type

relation_logits = relation_classifier(attended_features)

relation_type, confidence = softmax(relation_logits)

IF confidence >= RELATION_CONFIDENCE_THRESHOLD: # 0.6

relation = {

'subject': subject,

'predicate': relation_type,

'object': object,

'confidence': confidence

}

relations.APPEND(relation)

# Step 4: Confidence Assessment and Quality Control

knowledge_triples = []

FOR EACH relation IN relations:

# Calculate composite confidence score

conf_score = (

w1 semantic_similarity(relation, knowledge_base) +

w2 frequency_score(relation) +

w3 source_credibility(relation)

)

IF conf_score >= 0.6:

knowledge_triples.APPEND(relation)

ELSE:

# Queue for human verification (5% of cases)

human_verification_queue.APPEND(relation)

# Step 5: Human-in-the-Loop Validation (for low-confidence cases)

verified_triples = human_expert_review(human_verification_queue)

knowledge_triples.EXTEND(verified_triples)

RETURN knowledge_triples

END FUNCTION

# Algorithm 2: Heterogeneous Data Fusion

FUNCTION HeterogeneousDataFusion(data_sources):

INPUT: data_sources - Dict of {source_name: data, format: schema}

OUTPUT: unified_rdf_triples - Standardized RDF representation

# Step 1: Schema Mapping

mapping_rules = load_predefined_mappings() # MARC->RDF, DC->RDF, etc.

unified_triples = []

FOR EACH source, data IN data_sources.items():

IF source.format IN ['MARC21', 'Dublin Core', 'JSON']:

# Apply automatic mapping (70% coverage)

auto_mapped = apply_mapping_rules(data, mapping_rules[source.format])

unified_triples.EXTEND(auto_mapped)

ELSE:

# Complex mappings: human expert definition (30%)

expert_mapped = expert_schema_mapping(data, source.format)

unified_triples.EXTEND(expert_mapped)

# Step 2: Ontology Alignment

aligned_triples = ontology_alignment(

triples=unified_triples,

base_ontology='BIBFRAME_2.0',

custom_modules=['user_behavior', 'discipline_knowledge', 'academic_resources']

)

# Step 3: Cross-Validation (15% sample)

sample = random_sample(aligned_triples, ratio=0.15)

validation_accuracy = expert_validation(sample)

IF validation_accuracy >= 0.98:

RETURN aligned_triples

ELSE:

# Refine mapping rules and retry

refined_rules = refine_mappings_based_on_errors(sample)

RETURN HeterogeneousDataFusion(data_sources, refined_rules)

END FUNCTION

# Algorithm 3: Knowledge Graph-Based Personalized Recommendation

FUNCTION PersonalizedRecommendation(user_id, knowledge_graph):

INPUT: user_id - User identifier

knowledge_graph - KG with entities and relations

OUTPUT: recommended_items - Ranked list of recommendations

# Step 1: Build User Profile

user_history = get_user_interactions(user_id) # borrowed, accessed, searched

user_interests = extract_interests(user_history, knowledge_graph)

user_embedding = compute_user_embedding(user_interests)

# Step 2: Candidate Generation

candidate_items = []

FOR EACH interest IN user_interests:

# Find semantically related items via KG paths

related = knowledge_graph.traverse(

start=interest,

max_depth=5, # 5-hop reasoning

relation_types=['similar_to', 'cited_by', 'belongs_to_discipline']

)

candidate_items.EXTEND(related)

# Step 3: Ranking via Embedding Similarity

ranked_items = []

FOR EACH item IN candidate_items:

item_embedding = knowledge_graph.get_embedding(item)

similarity = cosine_similarity(user_embedding, item_embedding)

ranked_items.APPEND((item, similarity))

# Sort by similarity score (descending)

ranked_items.SORT(key=lambda x: x[1], reverse=True)

# Step 4: Path-Based Explainability

top_recommendations = []

FOR EACH item, score IN ranked_items[:10]:

# Find shortest path from user interests to recommended item

explanation_path = knowledge_graph.shortest_path(

source=user_interests[0],

target=item

)

# Calculate path probability

path_prob = PRODUCT(

transition_probability(edge) FOR edge IN explanation_path

)

recommendation = {

'item': item,

'relevance_score': score,

'explanation_path': explanation_path,

'path_confidence': path_prob

}

top_recommendations.APPEND(recommendation)

RETURN top_recommendations

END FUNCTION

# Algorithm 4: Knowledge Graph Construction and Storage

FUNCTION ConstructKnowledgeGraph(triples, storage_backend='Neo4j'):

INPUT: triples - List of validated (subject, predicate, object) triples

storage_backend - Graph database system

OUTPUT: knowledge_graph - Queryable graph structure

# Initialize graph database connection

graph_db = connect_to_database(storage_backend)

# Step 1: Create Entities (Nodes)

entities_created = SET()

FOR EACH triple IN triples:

FOR entity IN [triple.subject, triple.object]:

IF entity NOT IN entities_created:

node = graph_db.create_node(

label=entity.type,

properties={

'id': entity.id,

'label': entity.label,

'embedding': compute_embedding(entity),

'metadata': entity.metadata

}

)

entities_created.ADD(entity)

# Step 2: Create Relations (Edges)

FOR EACH triple IN triples:

graph_db.create_relationship(

source=triple.subject.id,

target=triple.object.id,

relation_type=triple.predicate,

properties={

'confidence': triple.confidence,

'timestamp': current_time(),

'source': triple.data_source

}

)

# Step 3: Build Indexes for Fast Retrieval

graph_db.create_index(entity_type='Book', property='title')

graph_db.create_index(entity_type='Author', property='name')

graph_db.create_index(relation_type='borrowed', property='timestamp')

# Step 4: Compute Graph Embeddings

graph_embeddings = graph_embedding_model.fit(graph_db, method='GraphSAGE')

FOR EACH node IN graph_db.all_nodes():

node.embedding = graph_embeddings[node.id]

RETURN graph_db

END FUNCTION
