# Supplementary material for: Knowledge graph-based intelligent data management and information innovation service model for university library systems
Source: PLoS One. 2026 Jan 16;21(1):e0341307. doi: 10.1371/journal.pone.0341307 (PMC12810841; doi:10.1371/journal.pone.0341307)
Supplement: S4 File — (DOCX) [file pone.0341307.s004.docx]

Supplementary File S4: Model Hyperparameters and Training Configuration

# Model Hyperparameters and Training Configuration

## 1. BERT-Based Named Entity Recognition (BERT-NER)

### Model Architecture

- Base Model: BERT-base-chinese (12 layers, 768 hidden size, 12 attention heads)

- Total Parameters: 110M

- Task Head: Token classification layer (768 → 17 classes)

- 17 classes = 8 entity types × 2 (BIO tagging) + 1 (O tag)

### Training Configuration

- Training Data: 200,000 manually annotated bibliographic records

- Validation Split: 80% training / 10% validation / 10% test

- Batch Size: 32

- Learning Rate: 2e-5 (with warmup)

- Optimizer: AdamW (weight_decay=0.01)

- Epochs: 5

- Max Sequence Length: 512 tokens

- Warmup Steps: 500

- Learning Rate Schedule: Linear decay

- Dropout Rate: 0.1

- Gradient Clipping: Max norm 1.0

### Performance Metrics (Test Set)

- Entity Recognition Precision: 94.2%

- Entity Recognition Recall: 91.5%

- Entity Recognition F1: 92.8%

### Hardware

- GPU: 4× NVIDIA A100 (40GB)

- Training Time: ~12 hours

- Inference Speed: ~150 sentences/second

---

## 2. Bi-LSTM with Attention for Relation Extraction

### Model Architecture

- Embedding Layer: 300-dimensional GloVe embeddings (pre-trained on library corpus)

- Bi-LSTM Layers: 2 layers

- Hidden size: 256 per direction (512 total)

- Dropout: 0.3

- Attention Mechanism: Multi-head attention (4 heads)

- Classification Head:

- Dense layer 512 → 128 (ReLU activation, dropout=0.3)

- Output layer 128 → 12 (relation types)

- Total Parameters: ~8.5M

### Training Configuration

- Training Data: 150,000 relation-annotated sentence pairs

- Validation Split: 80% / 10% / 10%

- Batch Size: 64

- Learning Rate: 0.001

- Optimizer: Adam (β1=0.9, β2=0.999)

- Epochs: 15

- Max Sequence Length: 100 tokens

- Loss Function: Categorical cross-entropy

- Early Stopping: Patience=3 epochs

### Performance Metrics

- Relation Extraction Precision: 90.3%

- Relation Extraction Recall: 89.1%

- Relation Extraction F1: 89.7%

### Hardware

- GPU: 2× NVIDIA V100 (32GB)

- Training Time: ~8 hours

- Inference Speed: ~500 sentence pairs/second

---

## 3. GPT-4 API for Complex Semantic Understanding

### API Configuration

- Model: gpt-4-0613

- Temperature: 0.3 (low for factual tasks)

- Max Tokens: 512

- Top-p Sampling: 0.9

- Frequency Penalty: 0.0

- Presence Penalty: 0.0

### Usage Strategy

- Trigger Condition: BERT-NER confidence < 0.7

- Usage Rate: ~8% of total data

- Cost: ~$0.03 per 1,000 tokens (GPT-4 pricing as of 2024)

- Total Cost for 100K records: Approximately $240 USD

### Prompts Used

Entity Disambiguation Prompt:

```

Given the following bibliographic text and an ambiguous entity mention,

determine the correct entity type and disambiguate if necessary.

Text: [CONTEXT]

Entity mention: [ENTITY]

Candidate types: Book, Author, Publisher, Discipline, Keyword, User, Journal, Conference

Return JSON: {"entity": "...", "type": "...", "confidence": 0.0-1.0}

```

Cross-lingual Entity Alignment Prompt:

```

Align the following Chinese and English bibliographic entries.

Determine if they refer to the same work.

Chinese: [CHINESE_ENTRY]

English: [ENGLISH_ENTRY]

Return JSON: {"is_match": true/false, "confidence": 0.0-1.0, "reason": "..."}

```

---

## 4. Graph Embedding Model (GraphSAGE)

### Model Architecture

- Framework: PyTorch Geometric 2.3.0

- Embedding Dimension: 128

- Number of Layers: 3

- Aggregator Function: Mean aggregator

- Neighborhood Sampling: [25, 10, 5] (per layer)

- Activation Function: ReLU

- Dropout: 0.5

### Training Configuration

- Loss Function: Unsupervised loss (negative sampling)

- Negative Samples: 20 per positive edge

- Batch Size: 512 nodes

- Learning Rate: 0.01

- Optimizer: Adam

- Epochs: 50

- Training Strategy: Inductive learning (generalizes to unseen nodes)

### Performance

- Link Prediction AUC: 0.89

- Node Classification F1: 0.86 (on downstream task)

- Embedding Quality: Validated via t-SNE visualization showing clear entity clusters

### Hardware

- GPU: 2× NVIDIA A100 (40GB)

- Memory Usage: ~35GB peak

- Training Time: ~6 hours for 2.5M nodes + 4M edges

- Inference Speed: ~10,000 nodes/second

---

## 5. Knowledge Graph Storage (Neo4j)

### Database Configuration

- Version: Neo4j Enterprise 5.9.0

- Storage Backend: Native graph storage

- Initial Heap Size: 16GB

- Max Heap Size: 32GB

- Page Cache Size: 20GB

- Transaction Log: Enabled (for ACID compliance)

### Indexing Strategy

- Label Indexes: Book(title), Author(name), User(id), Discipline(name)

- Relationship Indexes: borrowed(timestamp), cited_by(confidence)

- Full-text Search: Enabled on Book.title and Book.abstract

- Vector Index: For embedding-based similarity search (128-dim)

### Performance Metrics

- Query Response Time:

- Simple traversal (1-2 hops): <50ms

- Complex traversal (3-5 hops): 200-1500ms (95th percentile: 1.6s)

- Concurrent Users: Supports 1,200+ concurrent queries

- Throughput: 1,200 read queries/second, 600 write queries/second

---

## 6. Software Environment and Dependencies

### Core Frameworks

```

Python: 3.9.13

PyTorch: 2.0.1

Transformers (Hugging Face): 4.30.2

PyTorch Geometric: 2.3.0

Neo4j Python Driver: 5.9.0

spaCy: 3.5.3

pandas: 2.0.2

numpy: 1.24.3

scikit-learn: 1.2.2

```

### Hardware Infrastructure

- CPU: 2× Intel Xeon Gold 6248R (48 cores total)

- RAM: 256GB DDR4

- Storage: 10TB NVMe SSD RAID

- GPU: 4× NVIDIA A100 (40GB each)

- Network: 10 Gbps Ethernet

### Deployment

- Operating System: Ubuntu 22.04 LTS

- Containerization: Docker 24.0.2

- Orchestration: Kubernetes 1.27

- Load Balancer: Nginx 1.24

- Monitoring: Prometheus + Grafana
